# Statin use and breast cancer survival: a nationwide cohort study in Scotland

**DOI:** 10.1186/s12885-016-2651-0

**Published:** 2016-08-04

**Authors:** Úna C. Mc Menamin, Liam J. Murray, Carmel M. Hughes, Chris R. Cardwell

**Affiliations:** 1 Cancer Epidemiology and Health Services Research Group, Centre for Public Health, Queen’s University Belfast, Belfast, Northern Ireland; 2 School of Pharmacy, Queen’s University Belfast, Belfast, BT9 7BL Northern Ireland; 3 Centre of Excellence for Public Health (NI), Centre for Public Health, Queen’s University Belfast, Belfast, Northern Ireland; 4 Institute of Clinical Sciences Block B, Queen’s University Belfast, Royal Victoria Hospital, Belfast, BT12 6BA Northern Ireland

**Keywords:** Statins, Breast Cancer, Scotland, Pharmacoepidemiology

## Abstract

**Background:**

Preclinical evidence suggests that statins could delay cancer progression. Previous epidemiological findings have been inconsistent and some have been limited by small sample sizes, as well as certain time-related biases. This study aimed to investigate whether breast cancer patients who were exposed to statins had reduced breast cancer-specific mortality.

**Methods:**

We conducted a retrospective cohort study of 15,140 newly diagnosed invasive breast cancer patients diagnosed from 2009 to 2012 within the Scottish Cancer Registry. Dispensed medication usage was obtained from linkages to the Scottish Prescribing Information System and breast cancer-specific deaths were identified from National Records of Scotland Death Records. Using time-dependent Cox regression models, hazard ratios (HR) and 95 % confidence intervals (CI) were calculated for the association between post-diagnostic exposure to statins (including simvastatin) and breast cancer-specific mortality. Adjustments were made for a range of potential confounders including age at diagnosis, year of diagnosis, cancer stage, grade, cancer treatments received, comorbidities, socioeconomic status and use of aspirin.

**Results:**

A total of 1,190 breast cancer-specific deaths occurred up to January 2015. Overall, after adjustment for potential confounders, there was no evidence of an association between statin use and breast cancer-specific death (adjusted HR 0.93, 95 % CI 0.77, 1.12). No significant associations were observed in dose–response analyses or in analysis of all-cause mortality. For simvastatin use specifically, a weak non-significant reduction in breast cancer-specific mortality was observed compared to non-users (adjusted HR 0.89, 95 % CI 0.73, 1.08). Statin use before diagnosis was weakly associated with a reduction in breast cancer-specific mortality (adjusted HR 0.85, 95 % CI 0.74, 0.98).

**Conclusion:**

Overall, we found little evidence of a protective association between post-diagnostic statin use and cancer-specific mortality in a large nation-wide cohort of breast cancer patients. These findings will help inform the decision whether to conduct randomised controlled trials of statins as an adjuvant treatment in breast cancer.

## Background

Statins (3-hydroxy-3-methylglutaryl coenzyme A reductase inhibitors) are cholesterol-lowering drugs widely prescribed in the primary and secondary prevention of cardiovascular disease. Growing laboratory evidence suggests that statins may also have anti-cancer effects [1] through inhibition of cellular proliferation [2], induction of apoptosis [3] and suppression of tumour cell migration [4]. In breast cancer, the anti-proliferative effects of statins have been demonstrated both in vitro [5] and in vivo [6], and are particularly strong for lipophilic statins (such as simvastatin) [6]. Interestingly, preclinical studies of breast cancer have indicated that the reduction in cell proliferation may be more marked in oestrogen receptor (ER) − negative cells [5], suggesting that ER-negative tumours may be more sensitive to the potential anti-cancer effects of statins. Moreover, the antiproliferative and proapoptotic potential for statins have been demonstrated in breast cancer clinical trials of lipophilic [7, 8] and hydrophilic statins [9].

Statin use is associated with a reduction in cancer mortality [10] and thus far, a number epidemiological studies have investigated the association between statin use after breast cancer diagnosis and breast cancer-specific mortality (or recurrence). In a large Finnish study of newly diagnosed breast cancer patients, a significant 54 % reduction in breast cancer-specific mortality was observed, which was dose-dependent and similar for both hydrophilic and lipophilic statins [11]. This investigation however, against recommendations [12], used unlagged analyses (i.e. did not exclude medication use immediately prior to death which could be influenced by impending death), thus any observed protective association between statin use and breast cancer mortality could partly reflect not starting statin use, or the discontinuation of statin use, in fatally ill cancer patients [13]. There was also a lack of adjustment for important potential confounders such as other medication use and comorbidities [11]. A second study included breast cancer patients diagnosed in England and we previously reported a weak non-significant 16 % reduction in breast cancer death in statin users after diagnosis which appeared slightly more marked for the highly lipophilic statin simvastatin [14]. The relationship between statin use after diagnosis and breast cancer mortality however did not follow a clear dose–response and was attenuated in fully adjusted analyses [14]. In a large Danish cohort study of women diagnosed with stage I–III breast cancer, using as recommended, an exposure lag, a 20 % reduction in risk of cancer recurrence was observed among users of statins after diagnosis. The reduction in breast cancer recurrence was also observed in lipophilic statin users (adjusted HR 0.70, 95 % CI 0.53 to 0.92), whereas no association was observed for hydrophilic statins [15]. Other investigations of statin use and breast cancer recurrence have shown little evidence of associations [16, 17], however they were relatively small in size [16–18], and some [18] had potential for immortal time bias [19]. Despite previous studies not being conclusive, there have been calls for clinical trials [20]. Therefore, there is a need for additional well conducted population-based studies to inform the conduct of future clinical trials of statins (particularly simvastatin) in breast cancer patients. Therefore, in a large nation-wide study of newly diagnosed breast cancer patients, we aimed to evaluate the association between statin use after breast cancer diagnosis and breast cancer-specific mortality and to determine whether the association differs by drug solubility. We also aimed to determine if the association was modified by breast cancer estrogen receptor status.

## Methods

### Data sources

The study utilised linkages between national datasets from Scotland including the Scottish Cancer Registry (SMR06), the Prescribing Information System, the General/Acute Inpatient and Day Case dataset (SMR01), the Outpatient Attendance dataset (SMR00) and the National Records of Scotland Death Records. The Scottish Cancer Registry captures information on all cancers occurring in Scotland including date and site of primary cancer diagnosis, stage, grade and treatment data. The Prescribing Information System (available from January 2009 to January 2015) holds all medicines dispensed in the community in Scotland. The General/Acute Inpatient and Day Case dataset (available from January 1999 to January 2015) contains information on hospital diagnoses and operations and the Outpatient Attendance dataset (available from January 1999 to January 2015) contains diagnosis and procedures from new and follow up appointments at outpatient clinics. The National Records of Scotland Death Records contain date and cause of death up to January 2015. Linkages between data sources were conducted using the Community Health Index number (a unique number unique to individuals in Scotland).

### Study population

A cohort of newly diagnosed invasive breast cancer patients was identified on the basis of a Scottish Cancer Registry recorded primary diagnosis of breast cancer (ICD code C50) between January 2009 and December 2012. Cohort members with previous Scottish Cancer Registry cancer diagnosis (after January 1999), apart from in situ neoplasms and non-melanoma skin cancers, were excluded. Deaths were identified from National Records of Scotland with coverage up to 1st January 2015 (or from Scottish Cancer Registry death records) with breast cancer-specific deaths defined as those with breast cancer as the underlying cause of death (ICD code C50). Deaths in the first year after breast cancer diagnosis were removed (sensitivity analysis was conducted varying this interval) as it is likely that these patients had stage IV disease and it seemed unlikely that short term post-diagnostic medication usage could influence such deaths, therefore follow-up started one year after diagnosis. The patients were followed from one year after breast cancer diagnosis to death, the date they left Scotland or 1st January 2015.

### Study design

#### Exposure data

Statins dispensed in the community (identified from the Prescribing Information System) consisted of all medications in the Statins section of the British National Formulary (BNF) [21] (Section 2.12). A quantity of 28 tablets was assumed for the less than 0.1 % of dispensed prescriptions where quantity was assumed incorrect. Statin use was investigated as a time varying covariate [19] (patients were initially considered non-users and then users after a lag of 6 months after their first statin prescription). The use of a lag is recommended [12] and was used to exclude prescriptions in the six months prior to death as these may reflect changes due to end of life care (in sensitivity analyses the duration of this lag was varied). Medications may be withdrawn from cancer patients in whom death is suspected to be imminent and unlagged time-varying covariate analysis can bias results due to reverse causality [13]. Dose–response analyses were conducted with individuals considered non-users prior to 6 months after first use, a short term user between 6 months after first use and 6 months after the 12th prescription and a longer term user after this time. Further analyses was conducted by drug solubility including lipophilic (simvstatin and fluvastatin) and hydrophilic (atorvastatin, rosuvastatin and pravastatin) statins. Separate analysis investigated the influence of simvastatin use compared to non-use of simvastatin.

### Covariates

Data available from the Scottish Cancer Registry included AJCC cancer stage [22], histological grade (grade 1, 2 or 3), hormone receptor status (including estrogen, progesterone and HER2 status) and cancer treatments (including surgery, chemotherapy and radiotherapy in the six months after diagnosis). Comorbidities that contribute to the Charlson index were determined prior to diagnosis based upon ICD10 diagnosis codes, as described previously [23], in Scottish hospital inpatient (SMR01) and outpatient data (SMR00). A measure of socioeconomic status measure was determined using the 2009 Scottish Index of Multiple Deprivation based upon postcode of residence [24] which comprises super output area (SOA) level measures of multiple deprivation (based on residential postcodes) and is made up of seven SOA level domain indices. Information on hormone therapy use (including tamoxifen and aromatase inhibitor) was obtained from dispensed prescription records. Similarly, low-dose aspirin use, metformin and hormone replacement therapy (HRT) use (either estrogen alone or combination) were determined from dispensing records.

### Statistical analysis

In the main analysis, time-dependent Cox regression models were used to calculate hazard ratios for breast cancer-specific death (HRs) and 95 % confidence intervals (95 % CIs) for post-diagnostic statin users (regardless of pre-diagnostic statin use) compared with non-users using a time varying covariate as described previously. Adjusted analyses were conducted including the following potential confounders: age at diagnosis (continuous), year of diagnosis (in 1-year bands), socioeconomic status (in fifths), grade (1, 2 or 3), stage (1, 2, 3 or 4), surgery (yes or no within 6 months), radiotherapy (yes or no within 6 months), chemotherapy (yes or no within 6 months), aromatase inhibitor (yes or no as a time varying covariate), tamoxifen (yes or no as a time varying covariate), comorbidities (yes or no prior to diagnosis, including acute myocardial infarction, congestive heart failure, peripheral vascular disease, cerebral vascular accident, pulmonary disease, peptic ulcer, liver disease, diabetes, renal disease), hormone replacement therapy use (yes or no in year prior to diagnosis), aspirin and metformin usage (yes or no after post-diagnosis, as time varying covariates). Analyses were conducted by number of dispensed statin prescriptions and repeated for all-cause mortality. Subgroup analyses were conducted by cancer stage, year of diagnosis and estrogen receptor status. Separate sensitivity analysis was conducted by additionally adjusting for tumour hormone receptor status and increasing the lag from six months to 1 year, thereby excluding prescriptions in the year prior to death. A simplified analysis was also conducted using Cox regression to compare statin users to statin non-users in the first year after breast cancer diagnosis in individuals living more than 1 year after diagnosis; this controls immortal time bias [25] without requiring time varying covariates. An analysis was conducted based upon statin prescriptions in the year prior to diagnosis, regardless of post-diagnostic statin use (excluding patients diagnosed in 2009 for whom a full year of prescription records prior to diagnosis may not be available), not excluding deaths in the first year after diagnosis. An adjusted analysis for pre-diagnostic statin use was first conducted omitting stage, grade, cancer treatment from adjustments for potential confounders to avoid over-adjustment [26, 27], as these could be on the causal pathway for breast cancer-specific mortality. A separate analysis was also conducted using the time varying covariate approach with breast cancer-specific death as the outcome, adjusting for the competing risk of deaths from other causes, using competing-risks regression based on Fine and Gray’s proportional subhazards model [28]. Similar sensitivity analyses were carried out for all-cause mortality. A further analysis was conducted for only cardiovascular deaths (where the underlying cause of death was ICD 10 codes I0-99, G45, Q20-26, F01 or equivalent ICD-9 codes) and for all deaths excluding these cardiovascular deaths.

## Results

### Patient cohort

A total of 15,140 newly diagnosed breast cancer patients without a prior cancer history and with at least one year of follow-up were identified for inclusion, in which there was on average 4 years of follow-up after diagnosis (sd = 1, minimum = 1, maximum = 6 years). Patient characteristics by statin use are shown in Table 1. Statin users were more likely to be older and to have a lower socioeconomic status. Stage and grade were generally similar by statin use, but a slightly smaller proportion of statin users compared with non-users had poorly differentiated tumours (31 % versus 35 %, respectively). Statin users were less likely to receive surgery, chemotherapy and tamoxifen; however they were more likely to receive radiotherapy and aromatase inhibitors. A greater proportion of statin users compared to non-users had comorbidities (particularly for cerebrovascular disease, diabetes and myocardial infarction) and were users of low-dose aspirin and metformin.

**Table 1:** Characteristics of breast cancer patients by statin use

	Statin use in the year prior to diagnosis^a^					Statin use in the year after diagnosis^b^				
	User		Non-user			User		Non-user		
	*n*	%	*n*	%		*n*	%	*n*	%	
	[*n* = *3*,*031*]		[*n* = *9*,*200*]			[*n* = *4*,*233*]		[*n* = *10*,*907*]		
Year of diagnosis										
2009						861	23.8	2,811	24.4	<0.001
2010	994	32.8	2,990	32.5	0.73	915	25.2	2,811	24.4	
2011	1,005	33.2	3,090	33.6		920	25.4	2,929	25.4	
2012	1,032	34.0	3,120	33.9		928	25.6	2,965	25.7	
Age at diagnosis										
<50	69	2.3	2098	22.8	<0.01	93	2.4	2,741	23.9	<0.001
50–59	322	10.6	2,516	27.3		447	12.3	3,246	28.2	
60–69	989	32.6	2,365	25.7		1,264	34.9	3,008	26.1	
70–79	911	30.1	1,242	13.5		1,082	29.9	1,508	13.1	
80–89	626	20.7	759	8.3		636	17.5	819	7.1	
≥90	114	3.8	220	2.4		102	2.8	194	1.7	
AJCC stage										
1	1086	35.8	3556	38.7	0.43	1400	38.6	4541	39.4	<0.001
2	1053	34.7	3415	37.1		1290	35.6	4421	38.4	
3	353	11.6	1033	11.2		425	11.7	1337	11.6	
4	208	6.9	492	5.3		141	3.9	443	3.8	
* Missing*	331	10.9	704	7.7		368	10.2	774	6.7	
Grade										
1	366	12.1	1089	11.8	<0.001	466	12.9	1363	11.8	0.003
2	1335	44	4008	43.6		1647	45.4	5139	44.6	
3	900	29.7	3172	34.5		1130	31.2	4125	35.8	
* Missing*	430	14.2	931	10.1		381	10.5	889	7.7	
Socioeconomic status										
1 (most deprived)	676	22.3	1419	15.4	<0.001	776	21.4	1,736	15.1	<0.001
2	709	23.4	1682	18.3		825	22.8	2,089	18.1	
3	582	19.2	1,914	20.8		723	20.0	2,391	20.8	
4	587	19.4	2,025	22.0		703	19.4	2,582	22.4	
5 (least deprived)	477	15.7	2,159	23.5		597	16.5	2,717	23.6	
Treatment (within 6 months)										
Surgery	2,163	71.4	7,322	79.6	<0.001	2,808	77.5	9,592	83.3	<0.001
Radiotherapy	1,219	40.2	3,278	35.6	<0.001	1,486	41.0	4,080	35.4	<0.001
Chemotherapy	546	18.0	3,566	38.7	<0.001	705	19.5	4,722	41.0	<0.001
Comorbidity prior to diagnosis										
Acute myocardial infarction	227	7.5	63	0.7	<0.001	246	6.8	59	0.5	<0.001
Congestive heart failure	144	4.8	96	1.0	<0.001	149	4.1	76	0.7	<0.001
Peripheral vascular disease	105	3.5	46	0.5	<0.001	114	3.1	50	0.4	<0.001
Cerebral vascular accident	282	9.3	120	1.3	<0.001	287	7.9	116	1.0	<0.001
Pulmonary disease	288	9.5	449	4.9	<0.001	305	8.4	499	4.3	<0.001
Petptic ulcer	73	2.4	100	1.1	<0.001	86	2.4	119	1.0	<0.001
Liver disease	8	0.3	13	0.1	0.34	8	0.2	17	0.1	0.16
Diabetes	395	13.0	115	1.3	<0.001	422	11.6	129	1.1	<0.001
Renal disease	101	3.3	94	1.0	<0.001	100	2.8	85	0.7	<0.001
Medication use in year after diagnosis										
Low-dose aspirin^c^	1,406	46.4	561	6.1	<0.001	1,617	44.6	737	6.4	<0.001
Aromatase inhibitors	1,840	60.7	3,658	39.8	<0.001	2,258	62.3	4,550	39.5	<0.001
Tamoxifen	850	28.0	4,038	43.9	<0.001	1,080	29.8	5,262	45.7	<0.001
Metformin	1,201	33.1	1183	10.3	<0.001	440	14.5	98	1.1	<0.001

^a^Analysis includes breast cancer patients who have more than 1 year of records prior to diagnosis and excludes patients diagnosed 2009 for whom full medication records not available in the year prior to diagnosis. ^b^Post-diagnostic statin use in the year after diagnosis among breast cancer patients who lived more than 1 year after diagnosis. ^c^Low-dose aspirin use in year after diagnosis for statin use in year after diagnosis columns, low-dose aspirin use in year prior to diagnosis for statin use in year prior to diagnosis columns

### Association between statin use after diagnosis and survival

The main findings are displayed in Table 2. After adjustment for potential confounders, there was little evidence of an association between statin use after diagnosis (regardless of pre-diagnostic use) and breast cancer-specific mortality (adjusted HR 0.95, 95 % CI 0.79, 1.15). There was also no evidence of a dose–response association when post-diagnostic exposure was investigated by increasing number of prescriptions after diagnosis. For post-diagnostic simvastatin use specifically, a weak non-significant reduction in breast cancer-specific mortality was observed compared to non-users of simvastatin (adjusted HR 0.89, 95 % CI 0.73, 1.08), Table 2. Results were similar for use of any lipophilic statin after diagnosis compared to non-use (adjusted HR 0.90, 95 % CI 0.74,1.11) and were slightly less marked for users of hydrophilic statins after diagnosis compared to non-users (adjusted HR 0.97, 95 % CI 0.76, 1.24). In analysis of all-cause mortality, statin use was weakly associated with a (non-significant) reduction in mortality in adjusted analysis (adjusted HR 0.88, 95 % CI 0.76, 1.01); however no dose–response relationship was observed by increasing number of prescriptions, Table 2. Results were similar for use of lipophilic statins, including simvastatin (adjusted HR 0.87, 0.75, 1.01), and hydrophilic statin use after diagnosis, Table 2.

**Table 2:** Association between statin use after diagnosis and breast cancer-specific and all-cause mortality in patients with breast cancer

	Breast cancer mortality	Patients	Person years	Age-adjusted HR (95 % CI)	*P*	Adjusted^a^ HR (95 % CI)	*P*
				[*n* = *15*,*140*]		[*n* = *13*,*060*]	
Breast cancer mortality							
Statin non-user	855	10,907	30,852	1.00 (ref. cat.)		1.00 (ref. cat.)	
Statin user	335	4,233	10,421	0.87 (0.76,0.99)	0.04	0.95 (0.79,1.15)	0.62
1-12 prescriptions	201	1,358	5,713	0.91 (ref. cat.)	0.22	0.94 (0.75,1.18)	0.61
≥12 prescriptions	134	2,875	4,708	0.82 (0.68,0.99)	0.04	0.97 (0.75,1.25)	0.79
Simvastatin non-user	966	12,115	33,845	1.00 (ref. cat.)		1.00 (ref. cat.)	
Simvastatin user	224	3,025	7,428	0.82 (0.71,0.95)	0.01	0.89 (0.73,1.08)	0.24
Lipophilic non-user	964	12,110	33,836	1.00 (ref. cat.)		1.00 (ref. cat.)	
Lipophilic user	226	3,030	7,437	0.83 (0.72,0.96)	0.01	0.90 (0.74,1.11)	0.33
Hydrophilic non-user	1,062	13,434	37,389	1.00 (ref. cat.)		1.00 (ref. cat.)	
Hydrophilic user	128	1,706	3,884	0.95 (0.79,1.14)	0.57	0.97 (0.76,1.24)	0.82
All-cause mortality							
Statin non-user	1,323	10,907	30,852	1.00 (ref. cat.)		1.00 (ref. cat.)	
Statin user	684	4,233	10,421	0.95 (0.86,1.04)	0.27	0.88 (0.76,1.01)	0.07
1–12 prescriptions	365	1,358	5,713	0.92 (0.82,1.04)	0.20	0.86 (0.73,1.02)	0.08
≥12 prescriptions	319	2,875	4,708	0.98 (0.86,1.11)	0.74	0.90 (0.75,1.08)	0.27
Simvastatin non-user	1,527	12,115	33,845	1.00 (ref. cat.)		1.00 (ref. cat.)	
Simvastatin user	480	3,025	7,428	0.93 (0.84,1.04)	0.21	0.87 (0.75,1.00)	0.06
Lipophilic non-user	1,525	12,110	33,836	1.00 (ref. cat.)		1.00 (ref. cat.)	
Lipophilic user	482	3,030	7,437	0.94 (0.85,1.04)	0.23	0.87 (0.75,1.01)	0.072
Hydrophilic non-user	1,764	13,434	37,389	1.00 (ref. cat.)		1.00 (ref. cat.)	
Hydrophilic user	243	1706	3884	0.94 (0.83,1.08)	0.41	0.93 (0.77,1.11)	0.40

^a^Model contains age, year of diagnosis, socioeconomic status, stage, grade, cancer treatment within 6 months (radiotherapy, chemotherapy, surgery), comorbidities (prior to diagnosis, including acute myocardial infarction, congestive heart disease, peripheral vascular disaese, cerebral vascular accident, pulmonary disease, peptic ulcer, liver disease, diabetes, renal disease), hormone replacement therapy use (in year prior to diagnosis), and other prescription medication use (tamoxifen, aromatase inhibitor and low-dose aspirin and metformin, as time varying covariates)

### Association between statin use before diagnosis and survival

Results for statin use in the year preceding diagnosis are shown in Table 3. In adjusted models, statin use before diagnosis (regardless of post-diagnostic use) was weakly associated with a reduction in breast cancer-specific mortality (adjusted HR 0.85, 95 % CI 0.74, 0.98). Results were similar for users simvastatin after diagnosis compared to non-users of simvastatin (adjusted HR 0.87, 95 % CI 0.75, 1.02) and in analysis of any lipophilic statin use (adjusted HR 0.88, 95 % CI 0.76, 1.02), Table 3. Results were slightly attenuated in analysis of hydrophilic statins use compared to non-use (adjusted HR 0.90, 95 % CI 0.75, 1.09). For all-cause mortality, a significant reduction in risk was observed in adjusted models (adjusted HR 0.75, 95 % CI 0.67, 0.84) and results were similar for simvastatin use compared to non-use (adjusted HR 0.83, 95 % CI 0.74, 0.92), Table 3.

**Table 3:** Association between statin use before diagnosis and breast cancer-specific and all-cause mortality in patients with breast cancer

	Breast cancer mortality	Patients	Person years	Age-adjusted HR (95 % CI)	*P*	Adjusted^a^ HR (95 % CI)	*P*
				[*n* = *12*,*231*]		[*n* = *12*,*230*]	
Breast cancer mortality							
Statin non-user	834	9,200	29,298	1.00 (ref. cat.)		1.00 (ref. cat.)	
Statin user	381	3,031	9,125	0.98 (0.86, 1.11)	0.71	0.85 (0.74,0.98)	0.03
Simvastatin non-user	963	10,175	32,229	1.00 (ref. cat.)		1.00 (ref. cat.)	
Simvastatin user	252	2,056	6,193	0.95 (0.83,1.09)	0.48	0.87 (0.75,1.02)	0.08
Lipophilic non-user	961	10,168	32,212	1.00 (ref. cat.)		1.00 (ref. cat.)	
Lipophilic user	254	2063	6,211	0.96 (0.83,1.10)	0.54	0.88 (0.76,1.02)	0.09
Hydrophilic non-user	1077	11,161	35,181	1.00 (ref. cat.)		1.00 (ref. cat.)	
Hydrophilic user	138	1,070	3,242	1.02 (0.85,1.21)	0.87	0.90 (0.75,1.09)	0.28
All-cause mortality							
Statin non-user	1,340	9,200	29,298	1.00 (ref. cat.)		1.00 (ref. cat.)	
Statin user	691	3,031	9,125	0.96 (0.88,1.06)	0.41	0.75 (0.67,0.84)	<0.001
Simvastatin non-user	1,567	10,175	32,229	1.00 (ref. cat.)		1.00 (ref. cat.)	
Simvastatin user	464	2,056	6,192	0.95 (0.86,1.06)	0.38	0.83 (0.74,0.92)	0.001
Lipophilic non-user	1,564	10,168	32,212	1.00 (ref. cat.)		1.00 (ref. cat.)	
Lipophilic user	467	2063	6211	0.96 (0.86,1.06)	0.42	0.83 (0.75,0.93)	0.001
Hydrophilic non-user	1,788	11,161	35,181	1.00 (ref. cat.)		1.00 (ref. cat.)	
Hydrophilic user	243	1,070	3,242	0.98 (0.86,1.12)	0.77	0.81 (0.70,0.93)	0.002

^a^Model contains age, year of diagnosis, socioeconomic status, comorbidities (prior to diagnosis, including acute myocardial infarction, congestive heart disease, peripheral vascular disaese, cerebral vascular accident, pulmonary disease, peptic ulcer, liver disease, diabetes, renal disease) hormone replacement therapy use (in year prior to diagnosis), low-dose aspirin use and metformin (in year prior to diagnosis)

### Sensitivity\secondary analyses

Sensitivity\secondary analyses results are shown in Table 4. In comparison to the main analysis of statin use after diagnosis and breast cancer-specific mortality, stratification by cancer stage did not materially alter effect estimates. The observed associations for post-diagnostic statin use were similar in patients diagnosed with ER positive tumours and after additional adjustment for ER, PR and human epidermal growth factor 2 (HER2) status. There was a suggestion of a modest reduction in breast cancer-specific mortality risk in patients diagnosed in 2009–2010 (adjusted HR 0.77, 95 % CI 0.60, 0.98); however there was no evidence of a statistical interaction. Results for post-diagnostic statin use and breast cancer-specific mortality remained unchanged after increasing the lag from six months to 1 year and after adjustment for competing risks of death, Table 4. There were also no marked differences in associations when analysis was repeated in a simple analysis of any statin use (or simvastatin use) compared to non-use in the year after diagnosis. For all-cause mortality, similar estimates were produced across a number of sensitivity analyses, Table 4. Similar results were also obtained in analyses of cardiovascular and non-cardiovascular deaths.

**Table 4:** Sensitivity analysis of association between statin use and breast cancer-specific and all-cause mortality in patients with breast cancer

	Medication user			Medication non-user			Age-adjusted HR (95 % CI)	*P*	Adjusted HR (95 % CI)	*P*	P interaction
	Cancer\all mortality	Patients	Person years	Cancer\all mortality	Patients	Person years					
Cancer-specific mortality											
Subgroup analyses: Statin users versus non-users^a^											
Stage 1–2	111	3,185	8,085	310	8,467	24,768	0.86 (0.69,1.08)	0.21	0.91 (0.68,1.21)	0.51	
Stage 1–3	209	3,671	9,224	555	10,793	28,147	0.91 (0.77,1.08)	0.29	1.02 (0.83,1.26)	0.85	
Diagnosed 2009–2010	207	2,187	6,820	569	5,211	19,746	0.79 (0.67,0.93)	0.06	0.77 (0.60,0.98)	0.04	0.08
Diagnosed 2011–2012	128	2,046	3,601	286	5,696	11,106	1.03 (0.83,1.28)	0.77	1.35 (0.98,1.85)	0.06	
Estrogen receptor positive	243	3,655	9,079	552	8,995	25,600	0.88 (0.75,1.03)	0.11	0.92 (0.72,1.16)	0.48	
Estrogen receptor negative	87	514	1197	279	1710	4705	0.95 (0.73,1.23)	0.69	1.06 (0.76,1.48)	0.72	
Hormone receptors available^b^	212	2610	6193	550	6918	18682	0.89 (0.75,1.05)	0.18	0.98 (0.77,1.25)	0.89	
Using 1 year lag^c^	320	4067	9695	870	11073	31578	0.91 (0.80,1.04)	0.18	1.00 (0.78,1.28)	0.99	
Adjusted for competing risk of death^d^											
Statin	335	4,233	10,421	855	10,907	30,852	0.88 (0.78, 1.00)	0.07	0.98 (0.81, 1.19)	0.88	
Simvastatin	224	3,025	7,428	966	12,115	33,845	0.83 (0.72, 0.97)	0.02	0.91 (0.75, 1.12)	0.38	
Use in first year after diagnosis^e^											
Statin user versus non-user	318	3,624	9,555	872	11,516	31,718	0.89 (0.78,1.02)	0.10	0.99 (0.82,1.21)	0.95	
Simvastatin user versus non-user	210	2,470	6,569	980	12,670	34,704	0.86 (0.74,1.00)	0.05	0.95 (0.78,1.17)	0.65	
All-cause mortality											
Subgroup analyses: Statin users versus non-users^a^											
Stage 1–2	297	3,185	8,085	554	8,467	24,768	0.99 (0.85,1.14)	0.87	0.89 (0.74,1.06)	0.20	
Stage 1–3	437	3,671	9,224	859	10,793	28,147	0.98 (0.87,1.10)	0.72	0.91 (0.78,1.06)	0.24	
Diagnosed 2009–2010	456	2,187	6,820	871	5,211	19,746	0.94 (0.83,1.05)	0.26	0.79 (0.66,0.94)	0.01	0.38
Diagnosed 2011–2012	228	2,046	3,601	452	5,696	11,106	0.97 (0.82,1.14)	0.71	1.02 (0.79,1.30)	0.89	
Estrogen receptor positive	549	3,655	9,079	946	8,995	25,600	0.98 (0.88,1.09)	0.70	0.85 (0.72,1.01)	0.06	
Estrogen receptor negative	119	514	1197	324	1710	4705	0.99 (0.79,1.24)	0.92	1.00 (0.74,1.34)	0.98	
Hormone receptors available^b^ (and adjusted for)	400	2610	6193	806	6918	18682	0.95 (0.84,1.08)	0.43	0.89 (0.74,1.07)	0.23	
Using 1 year lag^c^	648	4067	9695	1359	11073	31578	0.97 (0.88,1.07)	0.54	0.91 (0.75,1.10)	0.33	
Cardiovascular deaths	142	4233	10421	132	10907	30852	3.21 (2.53,4.06)	<0.001	1.07 (0.73,1.55)	0.74	
Non-cardiovascular deaths	542	4233	10421	1191	10907	30852	1.36 (1.23,1.50)	<0.001	0.86 (0.74,1.00)	0.06	
Use in first year after diagnosis^e^											
Statin user versus non-user	649	3,624	9,555	1,358	11,516	31,718	0.97 (0.88,1.07)	0.55	0.91 (0.79,1.06)	0.23	
Simvastatin user versus non-user	445	2,470	6,569	1,562	12,670	34,704	0.96 (0.86,1.07)	0.47	0.89 (0.77,1.04)	0.15	

^a^Based upon main time varying covariate analysis adjusted model contains age, year of diagnosis, socioeconomic status, stage, grade, cancer treatment within 6 months (radiotherapy, chemotherapy, surgery), comorbidities (prior to diagnosis, including acute myocardial infarction, congestive heart disease, peripheral vascular disease, cerebral vascular accident, pulmonary disease, peptic ulcer, liver disease, diabetes, renal disease) hormone replacement therapy use (in year prior to diagnosis) and other prescription medication use (tamoxifen, aromatase inhibitor, low-dose aspirin, and metformin as time varying covariates)

^b^Model contains all variables in ^a^ along with estrogen, progesterone and HER2 receptor status, where available

^c^Statin use modelled as a time varying covariate with a 1 year lag. Model contains all variables in ^1^ with prescription medication use all modelled with a 1 year lag (tamoxifen, aromatase inhibitor, low-dose aspirin use and metformin)

^d^Reported estimates are subdistribution hazard ratios and 95 % CIs

^e^Model contains age, year of diagnosis, deprivation, stage, grade, cancer treatment within 6 months (radiotherapy, chemotherapy, surgery), comorbidities (prior to diagnosis, including acute myocardial infarction, congestive heart disease, peripheral vascular disease, cerebral vascular accident, pulmonary disease, peptic ulcer, liver disease, diabetes, renal disease) hormone replacement therapy use (in year prior to diagnosis) and other prescription medication use in the first year after diagnosis (tamoxifen, aromatase inhibitor, low-dose aspirin use and metformin)

## Discussion

In a large cohort of cancer registry confirmed breast cancer patients, we found little evidence of a reduction in breast cancer-specific mortality associated with statin use after diagnosis, however, our data could not rule out a weak protective effect of statins on breast cancer mortality, consistent with the confidence intervals observed (adjusted HR 0.95, 95 % CI 0.79, 1.15). Associations remained similar in analysis of dose–response and across a number of sub-group and sensitivity analyses.

Statin use has been associated with a 15–20 % reduction in all-cancer mortality [10, 29] but few studies have investigated the influence of statins on breast cancer-specifc mortality. Our main results for statin use after diagnosis are inconsistent with findings from a Finnish study [11] which observed a substantial reduction in breast cancer mortality risk with statin use after diagnosis (adjusted HR 0.46, 95 % CI 0.38, 0.55) in a large cohort of breast cancer patients, with similar findings observed in analysis of all-cause mortality and in analysis by statin type. Statin use however was not lagged in this study, thus, the reduction in risk observed with post-diagnostic statin use may be affected by the possible discontinuation, or lack of initiation, of statin use in some patients due to imminent death. The authors were also unable to adjust for potential confounding by other medication use in their analyses (such as HRT and low dose aspirin [30]), in addition to comorbidities. In all analyses, we lagged medication use, as recommended [12], after diagnosis by 6 months and this period was varied in sensitivity analysis. Our results are not inconsistent with our previous study of a large English cohort of breast cancer patients diagnosed with linkages to the Clinical Practice Research Datalink (CPRD) [14]. We previously reported a weak reduction in the rate of breast cancer-specific mortality with post-diagnosis statin use (adjusted HR 0.84, 95 % CI 0.68, 1.04) and simvastatin use (adjusted HR 0.79 95 % CI 0.63, 1.00) [14], but our results from Scotland are weaker for both overall use (adjusted HR 0.93, 95 % CI 0.77, 1.12) and simvastatin use (adjusted HR 0.87, 95 % CI 0.72, 1.07). The findings of a more marked (albeit non-significant) reduction in cancer mortality risk with simvastatin use after diagnosis are in line with mounting laboratory evidence suggesting the pleiotropic effects of statins may be limited to lipophilic statins. Lipophilic statins (such as simvastatin) have been shown to decrease cell proliferation as well as inhibit tumour growth in in vivo mouse mammary tumour models [6]. In both the English and Scottish studies however, clear dose–response associations for stain use and breast cancer mortality were not observed. A number of studies have investigated statin use in relation to breast cancer recurrence risk. Some [15, 18], but not all [16, 17] have reported moderate reductions in breast cancer recurrence risk with statin use after diagnosis. The largest of these studies, a Danish cohort of 18,769 women diagnosed with stage I–III breast cancer, observed a significant 20 % reduction in the risk of breast cancer recurrence risk with post-diagnostic statin use [15]. Interestingly, in analysis by statin solubility, the reduction in breast cancer recurrence was seen only for users of lipophilic statins (e.g. simvastatin) [15].

In secondary analysis, we investigated the influence of pre-diagnostic statin use in the year prior to diagnosis and found a weak protective association with breast cancer-specific mortality. Although comparable with results from some previous population-based studies which observed protective (although weak) effects against breast cancer mortality [31, 32], it is unclear how clinically useful these results are due to the difficulty of intervention during the pre-diagnostic period. This is one of the few epidemiologic studies to evaluate statin use and breast cancer progression according to hormone receptor status. We did not find any difference in associations by ER status and results were similar after adjustment for ER, PR and HER2 receptor status. This contrasts preclinical evidence suggesting that the anti-proliferative effect of statins may be stronger for ER-negative tumours [5, 33].

Our study had a number of strengths and limitations. We utilized data from a nationwide cancer registry to identify all incident breast cancers diagnosed in Scotland between 2009 and 2013 with follow-up of up to 6 years after diagnosis. Linkages to mortality registries allowed for robust identification of breast cancer deaths, although some misclassification of cause is possible. Evidence from methodological comparative studies suggest that risk estimates are unlikely to be greatly affected [34]. This study benefitted from available information on important clinical factors including cancer stage, treatments and comorbidities. In addition, availability of breast cancer hormone receptor status facilitated further sub-group analyses. Record linkage to national general practitioner (GP) dispensed prescription records provided detailed information on the timing of medication usage which permitted temporal relationships to be explored as well as investigation by statin type. Misclassification by over-the-counter use is limited in this study as statins are not available over-the-counter (OTC) in Scotland, apart from low dose 10 mg simvastatin which became available in 2004 [35]. Similarly, OTC use of low-dose aspirin is possible but previous investigation within the General Practice Research Database found that the majority of chronic aspirin use was captured by prescription records [36]. Furthermore, valid treatment risk estimates have been previously demonstrated when there is potential for over-the-counter medication usage [37]. We did not have information on medication adherence but results were similar in analysis of multiple dispensed prescriptions, in which adherence may be more likely. Unfortunately, we could not examine the influence of statins on breast cancer recurrence risk as recurrences were not routinely captured within the Scottish Cancer Registry. Finally, although we adjusted for a range of potential confounders, as with all observational studies, we cannot rule out residual confounding by unrecorded (e.g. body mass index) or incomplete variables (e.g. cancer stage).

## Conclusion

In a large nationwide study of cancer-registry confirmed breast cancer patients, we found little evidence of a protective association between statin use and cancer-specific mortality. These findings will help inform the decision whether to conduct randomised controlled trials of statins as an adjuvant treatment in breast cancer.

### Consent statement

Informed patient consent was not required for this study.

## Abbreviations

AJCC, American Joint Committee on Cancer; BNF, British National Formulary; CI, confidence interval; CPRD, clinical practice research datalink; ER, oestrogen receptor; GP, general practitioner; HER2, human epidermal growth factor receptor 2; HMGCR, (3-hydroxy-3-methylglutaryl coenzyme A reductase); HR, hazard ratio; RT, hormone replacement therapy; ICD, International classification of diseases; NHS, National Health Service; NSS, National Services Scotland; PR, progesterone receptor)
